# BMSC–HNC Interaction: Exploring Effects on Bone Integrity and Head and Neck Cancer Progression

**DOI:** 10.3390/ijms241914417

**Published:** 2023-09-22

**Authors:** Jonas Eichberger, Daniel Froschhammer, Daniela Schulz, Konstantin J. Scholz, Marianne Federlin, Helga Ebensberger, Torsten E. Reichert, Tobias Ettl, Richard J. Bauer

**Affiliations:** 1 Department of Oral and Maxillofacial Surgery, University Hospital Regensburg, 93053 Regensburg, Germany; jonas.eichberger@ukr.de (J.E.); daniel.froschhammer@stud.uni-regensburg.de (D.F.); daniela.schulz@ukr.de (D.S.); torsten.reichert@ukr.de (T.E.R.); tobias.ettl@ukr.de (T.E.); 2 Department of Oral and Maxillofacial Surgery, Center for Medical Biotechnology, University Hospital Regensburg, 93053 Regensburg, Germany; 3 Department of Conservative Dentistry and Periodontology, University Hospital Regensburg, 93053 Regensburg, Germany; konstantin.scholz@ukr.de (K.J.S.); marianne.federlin@ukr.de (M.F.); helga.ebensberger@ukr.de (H.E.)

**Keywords:** head and neck cancer, bone marrow-derived stromal cells, collagenase enzymes, MMP-1, MMP-9

## Abstract

In recent research, the tumor microenvironment has been shown to attract mesenchymal stromal cells (MSCs), which is of particular interest due to its implications for cancer progression. The study focused on understanding the interaction between bone marrow-derived MSCs (BMSCs) and head and neck cancer (HNC) cells. This interaction was found to activate specific markers, notably the osteogenic marker alkaline phosphatase and the oncogene Runx2. These activations corresponded with the release of collagenase enzymes, MMP9 and MMP2. To gain insights into bone resorption related to this interaction, bovine bone slices were used, supporting the growth of “heterogeneous spheroids” that contained both BMSCs and HNC cells. Through scanning electron microscopy and energy-dispersive X-ray (EDX) analysis, it was observed that these mixed spheroids were linked to a notable increase in bone degradation and collagen fiber exposure, more so than spheroids of just BMSCs or HNC cells. Furthermore, the EDX results highlighted increased nitrogen content on bone surfaces with these mixed clusters. Overall, the findings underscore the significant role of BMSCs in tumor growth, emphasizing the need for further exploration in potential cancer treatment strategies.

## 1. Introduction

Head and neck squamous cell carcinoma (HNSCC) ranks as the sixth most prevalent malignancy globally, with an alarming increase in incidence over the past decade, accounting for 644,000 new cases annually [1]. Despite advances in treatment, the 5-year survival rate remains a dismal 50% [2]. Oral cancer, primarily driven by tobacco and alcohol consumption, constitutes nearly half of these cases [3].

Oral cancer is notorious for its rapid invasive growth into adjacent anatomical structures, including lymph nodes, making local cervical or distant recurrences relatively common [4]. The prognosis for HNSCC is generally poor, with invasion and metastasis being major contributors to mortality [5]. Over 60% of patients initially present with an advanced stage, where adjacent tissues and lymph nodes are already infiltrated [6]. Oral cavity cancer frequently invades the mandible, necessitating partial or segmental surgical resection when bone involvement is suspected, significantly impacting patients’ quality of life due to cosmetic and functional impairments [7]. Bone invasion in head and neck cancer (HNC) is a significant concern, particularly with mandibular bone involvement which significantly impacts disease staging and treatment [8].

Several studies have explored the role of stromal cells in promoting HNC bone invasion. It has been suggested that cancer-associated fibroblasts and mesenchymal stem cells may facilitate HNC invasion through their interactions with tumor cells. This interaction between stromal cells and HNC cells in the invasive front of tumors has been implicated in tumor formation and metastasis [9,10,11]. The infiltrative potential of HNC tumors contributes to their aggressive nature and poor prognosis.

From a tumor biology standpoint, the molecular determinants underlying the variable patterns of mandibular invasion in oral squamous cell carcinoma (OSCC) remain enigmatic. Research continues to explore the role of the RANK/RANKL/OPG system’s imbalance, which may contribute to changes in the osteoclast and osteoblast activity affecting bone formation and degradation [12,13].

Additionally, matrix metalloproteinases (MMPs), a family of zinc endopeptidases, are involved in bone remodeling and homeostasis [14]. Notably, MMP-1, MMP-2, and MMP-9 mutations are implicated in OSCC invasiveness and metastasis [15,16]. Our previous work has shown that a spheroid mixture of BMSCs and HNC tumor cells induces osteogenic markers, such as alkaline phosphatase (ALP) and Runx2, and differentially expresses and secretes MMP-9, resulting in increased cell invasion. BMSCs, capable of differentiating into various cell lineages, are recruited to the tumor microenvironment in response to signals from cancer cells [17]. Attracted by inflammatory cytokines and growth factors, circulating BMSCs settle in the tumor stroma or become integrated into the tumor mass itself [18]. There have been a number of studies and clinical trials that have reported contradictory effects of transplanted MSCs on the metabolism of tumors. Although some studies report that transplanted MSCs inhibit the metabolic activity of tumors, the majority of studies demonstrate that MSC transplantation has stimulatory effects that promote the growth, metastasis, and transformation of tumors [19,20,21,22].

On the basis of our findings, we hypothesize that the synergy between bone marrow stromal cells and oral cancer cells might speed up the process of bone resorption and increase tumor invasion. This could leave certain structures exposed and vulnerable to abnormal cellular activities, which could then foster bone invasion due to the disruption in matrix arrangement and structural integrity that was caused by the disruption.

## 2. Results

Our recent findings demonstrated a significant increase in collagenase MMP-9 secretion in heterogeneous spheroids composed of bone marrow stromal cells (BMSCs) and HNC cells [11]. Given the aggressive bone infiltration often observed in head and neck tumors and the recurrence of tumors after partial jaw resection and fibula transplantation, we hypothesized that BMSC and tumor cell mixtures create favorable conditions for bone attachment and invasion.

To investigate the potential factors contributing to bone erosion and invasion, we examined whether increased protease secretion from BMSC/HNC mixtures might render bone structure more vulnerable. We seeded three HNC cell lines (D562, A253, and SCC25) as heterogeneous spheroid mixtures with BMSCs and as homogeneous spheroids on mildly etched bovine bone slices. After the spheroids spread and covered most of the surface, the bone slices were cultured for a total of 3 weeks and subsequently analyzed using scanning electron microscopy.

**Figure 1:**
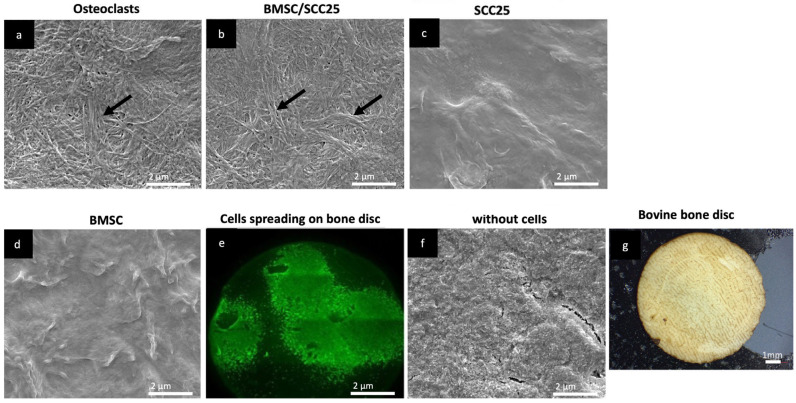
(**a**–**g**): scanning electron micrographs of bovine bone slices on which cells were cultured for 21 days. The exposure of collagen fibers depends on the cell type and cell mixture. Osteoclasts showed the strongest exposure of collagen fibers (highlighted with black arrows; (**a**) as well as mixtures of bone marrow stem cells and HNC cells, exemplified here with a mixture of BMSCs and SCC25 cells (**b**). Monocultures (**c**,**d**) and bone slices without cells showed much less exposure of fibers, in this example, none. After 21 days of seeding, spheroids spread on the bone slices. Here, spheroids are stained with Calcein AM, therefore resulting in optically visible green fluorescence ((**e**), see also Supplementary Figures S1 and S2). Unseeded bovine bone slice (**f**). Photograph of bovine bone disc (**g**). Dimensions indicated in each bar on the images. Bar = 2 µm.

In each case, four spheroids with 20,000 cells each were seeded. The cells were cultured on the bone slices for a total of 3 weeks. After this period all spheroids spread and covered most of the surface. Figure 1f shows the colonization of the bone slices with homogeneous and heterogeneous spheroids using Calcein-AM staining. The bone slices were analyzed by scanning electron microscopy, which revealed more or less strong exposures of collagen fibers, which we scored with three independent raters. As a positive control, osteoclasts were seeded onto the bones with a collagen exposure score of five (Figure 1).

**Figure 2:**
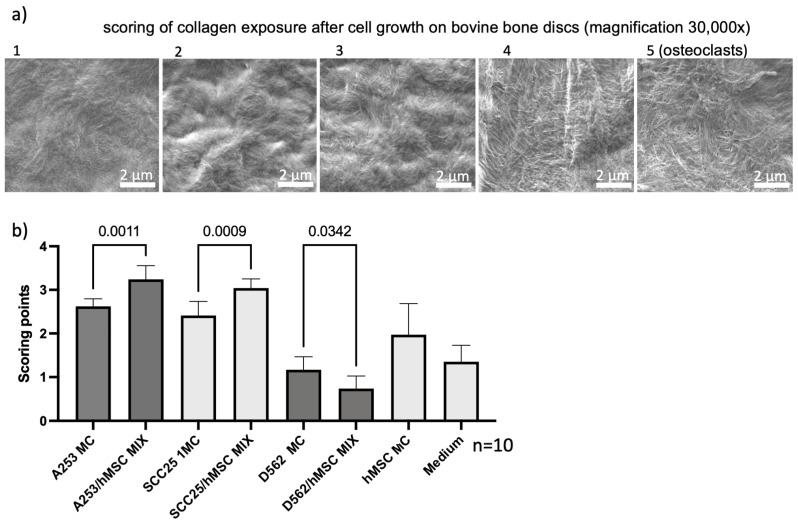
Visual scoring with 10 different samples each were performed independently and in a blinded manner by three raters. The scores were matched using the previously determined scoring table. Depending on the extent of collagen exposure, scoring numbers from 1–5 were assigned (**a**). The mixtures with bone marrow stem cells showed significantly increased scoring in two head and neck tumor cell lines, A253 and SCC25, respectively (**b**) (Supplementary Figure S2). Interestingly, the metastatic cell line D562 showed an opposite effect. After mixing, the scoring was significantly less. Scale bar = 2 µm.

Heterogeneous bone marrow stromal cell BMSC/HNC-mixed spheroids exhibited a notably increased collagen exposure for the A253 and SCC25 cell lines in comparison to their monoculture counterparts. Intriguingly, this observed pattern was inverted in the case of the metastatic D562 cell line, wherein mixed spheroids displayed a diminished degree of collagen exposure relative to homogeneous spheroids (Figure 2 and Figure S2).

**Figure 3:**
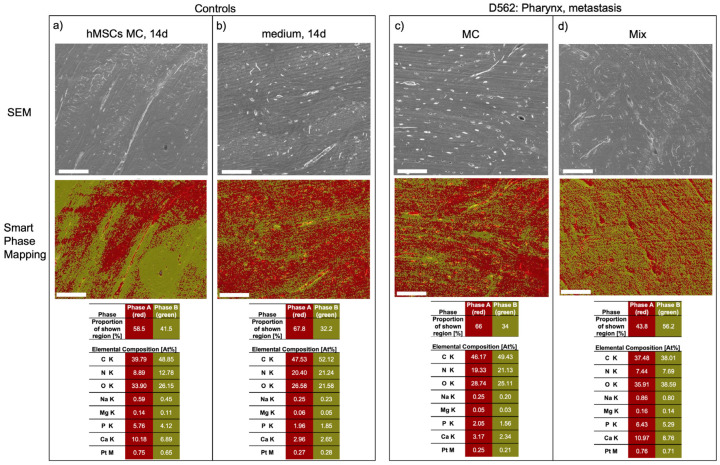
Exemplary SEM visualization and analysis of control groups, isolated BMSCs in monoculture (MC; (**a**)) and isolated cell medium (**b**); the metastatic cell line D562 showed similar results to the control groups in monoculture (MC) and BMSC/HNC mixtures (**c**,**d**). Upper row: high-vacuum SEM of a centrally located region on the surface of a randomly chosen bone specimen. Middle row: EDX smart phase mapping revealed two main phases (tolerance adjustment: 0.95) with a lower content of organic compounds represented by At%C and At%N and a higher content of inorganic compounds represented by At%Ca and At%P for Phase A (red) compared to Phase B (green). Bottom Row: Proportion of each identified phase as a percentage of all pixels of the corresponding region. Below, the relative elemental compositions of Phase A and B are depicted in relative atomic percent (At%) for each element. Platinum was present due to superficial sputtering of the specimens before SEM analysis. Scale bar = 100 µm.

EDX smart phase mapping supported our observations and data. Homogeneous spheroid cultures of A253 and SCC25 cell lines, as well as BMSC-only spheroids and the medium-only control, displayed two major phases with low inorganic content (phase A containing more At%C and At%N compared to phase B) and higher inorganic content (phase B containing more At%Ca and At%P compared to phase A). The metastatic cell line D562 exhibited similar results *regarding the elemental composition of different phases of the specimens* for both homogeneous and mixed spheroids (Figure 3).

**Figure 4:**
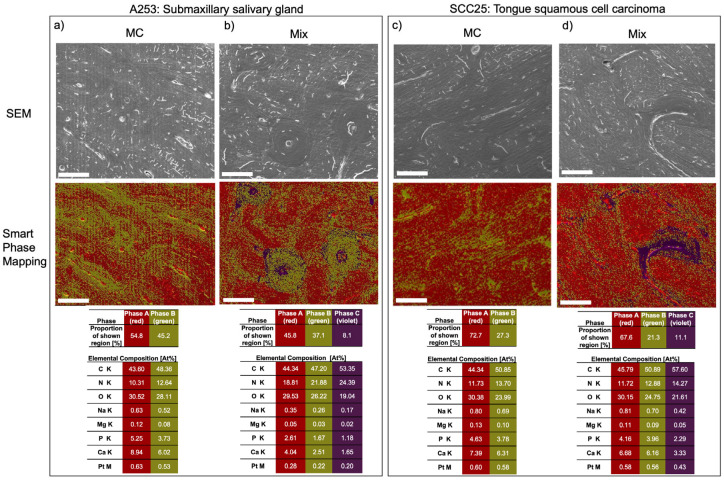
Exemplary SEM visualization and analysis of specimens following to exposure with HNC cells in monoculture (MC) (**a**,**c**) and in BMSC/HNC mixture of 10,000 cells each (**b**,**d**). Upper row: high-vacuum SEM of a centrally located region on the surface of a randomly chosen bone specimen; middle row: EDX smart phase mapping revealed a third main phase (Phase C, violet; tolerance adjustment: 0.95) showing the highest At%C and At%N of all phases in addition to Phase A and B, which were similar to Figure 3; phase C was detected mainly in areas morphologically matching the osteons ((**b**,**d**) second row) and may indicate areas of pronounced cell-induced demineralisation showing higher At%C and At%N and lower At%Ca and At%P possibly representing exposed collagen networks and mineral dissolution. The proportion of each identified phase as a percentage of all pixels of the corresponding region. Below, the relative elemental compositions of Phase A, B and C are depicted in atomic percent (At%) for each element. Platinum was present due to superficial sputtering of the specimens before SEM analysis. Scale bar = 100 µm.

Intriguingly, exemplary EDX analysis (smart phase mapping) of bone slices with heterogeneous BMSC/HNC mixtures for A253/BMSC and SCC25/BMSC revealed an additional third phase (phase C), characterized by the highest At%C (atomic percent carbon) and At%N (atomic percent nitrogen). Phase C was exclusively detected within the chosen experimental setting on bone slices cultured with heterogeneous spheroid mixtures, mainly in areas morphologically corresponding to the osteons (Figure 4b,d). These results suggest pronounced cell-induced demineralization, with higher At%C and At%N percentages and lower At%Ca and At%P percentages, possibly representing exposed collagen networks and mineral dissolution from the specimen surfaces.

## 3. Discussion

Our study aimed to explore the impact of BMSCs and HNC cell mixtures on bone material composition and potential tumor invasion. We found that heterogeneous spheroid mixtures of BMSCs and HNC cells, particularly for the A253 and SCC25 cell lines, resulted in more significant collagen exposure and bone demineralization than homogeneous spheroids. These findings suggest that the interaction between BMSCs and HNC cells may disrupt bone homeostasis, contributing to the invasive potential of oral cancer.

The percentage of HNC patients who show bone erosion or bone invasion of the tumor varies in different studies. Colnot et al. reported that about 40% of HNC patients had micrometastatic cells in the bone marrow [23]. Pichi et al. mentioned that bone metastasis occurred in approximately 22% of cases of advanced HNC [24]. Gibo et al. found that 40.3% of patients with OSCC had mandibular bone invasion [25]. A recent study suggested that 31.8% of patients with oral squamous cell carcinoma (OSCC) had mandibular invasion. Moreover, we also conducted a systematic review of other studies and found that the reported incidence of mandibular invasion in OSCC ranged from 12% to 56% [26].

The effect of bone marrow stem cells on the characteristics and functions of other cells, or vice versa, is well known in the literature. Amiri et al. investigates the effects of different bone marrow stromal cells (BMSCs) on the induction of quiescence and tested the advantage of pan-HDAC inhibitor panobinostat in the induction of apoptosis and targeting the quiescence cells of APL-derived (NB4) and CML-derived (K562) cell lines [27]. Moreover, Zhou et al. demonstrates that co-culture with leukemia cells stimulates the production of uPA, uPAR, PAI-1, MMP-9, and VEGF-A by BMSCs [28]. Liao et al. explored the effects of concentrated growth factors on the osteogenic differentiation of hBMSCs and human umbilical vein endothelial cells (HUVECs) in a co-culture system. The study found that 10% concentrated growth factor (CGF) had a significant osteogenic effect on human bone marrow mesenchymal stem cells (hBMSCs) directly co-cultured with human umbilical vein endothelial cells (HUVECs). The researchers suggest that autophagy activated by the mTOR/ULK1 pathway and CX43-mediated intercellular communication may play regulatory roles in promoting hBMSCs osteogenic differentiation [29].

In the present study, we focused our investigation on the observation of collagen exposure with particular HNC-derived cells in direct interaction with BMSCs. The results of our experiments suggest that HNC cells with specific primary sites of origin acquire an increased capacity to degrade, or expose, bone material when combined with BMSCs. In our experiments, it was striking that the single cell line D562, originally derived from a metastasis in the pharynx, did not show the same effects of bone erosion as the other two cell lines A253 and SCC15, whose primary sites of origin are the salivary gland and tongue. Of course, to prove this hypothesis, further studies need to be performed on a larger scale.

We have not provided direct evidence here that the markedly increased MMP-9 secretion of such a mixture [11,30], is also responsible for the effect observed here. However, based on a number of studies published by us and elsewhere in the literature indicating that MMPs play a role in bone invasion and erosion, it can be reasonably inferred that these proteases may have a major impact on bone erosion. At the very least, they could help create a niche for tumor cells to attach to bone in pre-existing propositions.

The role of MMPs in the invasiveness of OSCC has been well documented in the literature [31]. MMP-2 and MMP-9 are upregulated in most cancers and play crucial roles in modulating invasion and metastasis [32]. Recently, Eichberger et al. have demonstrated that loss of MMP-27 is a predictor of mandibular bone invasion in OSCC. The increased secretion of MMP-9 in BMSC/HNC mixtures observed in our studies [11,30] aligns with the findings of Li et al., who reported that higher levels of MMP-9 expression in ovarian cancer tissues are associated with tumor invasion and metastasis. They showed increased MMP-9 expression was related with a worse outcome in ovarian cancer patients (HR = 1.68, 95 percent CI 1.09–2.59, *p* = 0.02). Furthermore, MMP-9 expression in ovarian cancer was significantly higher than in non-malignant tumors (OR = 11.46, 95 percent CI 8.47–15.50, P0.00001). Furthermore, increased MMP-9 expression was associated with FIGO stage (OR = 4.85, 95 percent CI 2.60–9.04, P0.00001), grade of differentiation (OR = 3.34, 95 percent CI 2.46–4.54, P0.00001), lymph node metastasis (OR = 5.75, 95 percent CI 3.71–8.92, P0.00001), and there was no association with histological type of ovarian cancer [33]. The FIGO staging system is a globally recognized system used to classify the extent of spread of gynecological cancers, such as cervical, ovarian, and endometrial cancers.

Additionally, the RANK/RANKL/OPG system has been implicated in bone remodeling and metastasis in various cancers, including breast and prostate cancers [34]. In this context, our results provide further evidence supporting the involvement of this system in OSCC-related bone resorption.

The role of BMSCs in the tumor microenvironment is a topic of ongoing debate. The effects of MSCs on tumor growth and metastasis are complex and context dependent. MSCs can migrate to sites of injury, inflammation, and tumors, where they secrete various cytokines, chemokines, and growth factors that interact with the tumor microenvironment. The interaction between MSCs and malignant tumors provides new clinical ideas for treatment, but their effects on tumors are still inconclusive [35]. Liu et al. suggest that BMMSCs have a tumor-promoting effect on head and neck cancer (HNC) through Periostin-mediated phosphoinositide 3-kinase/Akt/mammalian target of rapamycin [36]. A study by Klopp et al. demonstrated that MSCs promote the growth of breast cancer cells in vivo, supporting the stimulatory effects of BMSCs on tumor growth and metastasis observed in our study [37]. In contrast, a study by Basmaeil et al. shows that MSCs have anti-tumor properties and can suppress tumor growth, regulate cellular signaling, and induce apoptosis in MDA231 cells in vitro [38].

Our results indicate that the impact of BMSC/HNC interactions may vary depending on the specific HNC cell line. This finding is in line with a study by Chen et al., who reported that the interactions between MSCs and various cancer cell lines have differential effects on tumor growth and invasion, highlighting the importance of considering the specific cellular context when investigating MSC functions in the tumor microenvironment [39].

In our study, we demonstrated that heterogeneous spheroid mixtures of BMSCs and HNC cells resulted in more significant collagen exposure and bone demineralization than homogeneous spheroids for the A253 and SCC25 cell lines. These results are in line with findings in the literature showing that cancer-associated fibroblasts (CAFs) and bone marrow-derived mesenchymal stem cells (BMSCs) promote bone invasion by cancer cells through the upregulation of matrix metalloproteinases (MMPs). BMSCs promote bone invasion by cancer cells through the upregulation of MMPs, which play a crucial role in the degradation of the ECM and are responsible for creating disturbances in ECM regulation, which is a prerequisite for unregulated tumor growth, local invasion, and metastasis [40,41].

This further supports the idea that stromal cells in the tumor microenvironment, including BMSCs, may contribute to bone degradation and tumor invasion.

Future research should focus on elucidating the molecular mechanisms underlying the differential effects of BMSC/HNC interactions on various HNC cell lines, as well as exploring the role of other stromal cells, such as CAFs, in OSCC progression. Additionally, investigating the potential therapeutic implications of these findings, such as the development of MMP inhibitors or targeting the RANK/RANKL/OPG pathway, could lead to novel treatment strategies for OSCC patients with bone invasion.

## 4. Materials and Methods

### 4.1. Cell Lines and Culture Conditions

The human head and neck cancer cell lines A-253 (submaxillary salivary gland of 54-year-old male patient), Detroit 562 (metastasis from a pharynx primary tumor, adult female patient), and SCC-25 (tongue, 70-year-old male patient) were ordered from ATCC as Head and Neck Panel TCP-1012^TM^. HNC cell lines were maintained in DMEM (PanBiotech, Aidenbach, Germany) supplemented with 10% fetal calf serum (FCS, Gibco, Carlsbad, CA, USA), 1% L-glutamine (Sigma-Aldrich, Munich, Germany) and 1% penicillin/streptomycin (Sigma-Aldrich, Munich, Germany) at 37 °C in a 5% CO_2_ humidified atmosphere. The medium was changed every two to three days and the cells were passaged prior reaching confluence. Detachment of the cells was achieved by incubation with 0.05% trypsin–EDTA solution (Sigma-Aldrich, Munich, Germany) for 5 to 10 min (min) at 37 °C.

BMSCs were provided by the Department of Experimental Orthopedics (ZMB, Asklepios Klinik, Bad Abbach, Germany). Stem cells were obtained from patients who underwent removal due to the installation of a total hip arthroplasty. The bone marrow was then aspirated, separated via density gradient centrifugation, and the resulting cells of mononuclear origin were expanded to passage 3 in MSC medium (Miltenyi Biotec, Bergisch Gladbach, Germany). The collected cells were regularly tested by flow cytometry for the following mesenchymal stem cell markers: CD44+, CD105+, CD90+, CD73+, CD34−, CD19−. Additionally, BMSCs were tested for their ability to differentiate into adipogenic, chondrogenic, and osteogenic lineages.

Osteoclasts were differentiated from THP-1 cells. For this purpose, THP-1 cells were seeded on both bone slices (Immunodiagnostic Systems Holdings PLC, Tyne and Wear, UK) and empty well plates after growing in a suspension culture with a cell count of 20,000 cells/well and 200 µL medium in a 96-well plate (Greiner Bio-One, Kremsmünster, Austria). In this culture, PMA (c = 100 ng/mL) was added to the conventional RPMI medium (Thermo Scientific, Waltham, MA, USA) to allow suspension cells to adhere. After 48 h, RANKL (c = 50 ng/mL) and hM-CSF (c = 50 ng/mL) were added to the adhered cells during the first media change to induce differentiation into osteoclasts.

### 4.2. Cultivation on Bone Slices

Bovine bone slices were purchased from Immunodiagnostic Systems Holdings PLC (Tyne and Wear, UK). Prior to use, the bone slices were etched with 50% citric acid for 10 s and equilibrated overnight (37 °C, 5% CO_2_) in 96-well culture plates in DMEM with 10% FCS, 1% penicillin/streptomycin. The bovine bone slices were placed in 96-well plates. For cultivation of spheroids on bone slices, 5 spheroids were placed on each bone slice and cultivated for 3 weeks in a 5%-CO_2_ humidified atmosphere. The medium was changed every 2–3 days, before the cultivated slices underwent further analyses.

### 4.3. Generation of Tumor Spheroids

For the experiments, tumor cell spheroids were used, which were cultivated by the “hanging drop method”. For this purpose, the cells were resuspended in the appropriate cultivation medium. After determining cell concentration, the desired amount of cell suspension, cellular components were centrifuged off, supernatant was aspirated and the cell pellet was resuspended in the desired concentration in the hanging-drop medium (2% FCS), 1% L-glutamine (200 mM), 1% penicillin/streptomycin, 0.5% EGF (1 µg/mL), 20% methocel stock solution (1.2% methylcellulose).

30 µL drops were pipetted onto the bottom of the lid of a Petri dish. To prevent drying, the bottom of the Petri dish was filled with 5 mL of PBS. The lids containing the cell suspensions were then carefully placed on the Petri dishes (Greiner Bio-One, Kremsmünster, Austria) and cultured at 37 °C, 5% CO_2_ in an incubator (Thermo Scientific) for 24 h.

Multicellular spheroids with cell numbers of 5000, 10,000, and 20,000 cells were generated at the time of seeding for the experimental approaches listed.

For the experimental approaches in which cells were to be cultured on bone slices, bovine bone slices cut to fit the 96-well plate were used.

### 4.4. Calcein-AM Staining

To determine the localization and spread of the cells on the bone slices, a detection with calcein-AM (Sigma-Aldrich, Munich, Germany) was made before detaching of the cells. Calcein-AM (3 µM, Sigma-Aldrich) was added to the medium and incubated for 30 min at 37 °C, 5% CO_2_. During this period, the calcein-AM is transported through the cell membrane into the cell where it is converted into calcein by esterase activity of the living cells. This forms a complex with calcium ions in the cytoplasm, resulting in optically visible green fluorescence.

Images were thus taken by fluorescent microscopy and heavily overgrown areas were marked for examination with the scanning electron microscope (Thermo Scientific, Waltham, MA, USA).

### 4.5. High Vacuum Scanning Electron Microscopic (HV-SEM) Examination

The bovine bone slices were fixed in glutaraldehyde (2.5% in Sörensen buffer pH7.4) for 30 min and subsequently washed with and stored in a Sörensen buffer pH 7.4 for 24 h at 4 °C. The buffer solution was removed using aqua dest. (3 × 5 min at 20 rpm, GFL 3018, Gesellschaft für Labortechnik mbH, Burgwedel, Germany). The samples were transferred to a 24-well cell culture plate (CELLSTAR, Cat.-No. 662160, Greiner Bio-One GmbH, Frickenhausen, Germany) and air dried for 16 h (37 °C; Heating cabinet, Memmert Typ U10, Memmert GmbH, Büchenbach, Germany). The specimens were mounted onto aluminum stubs (12.5 mm Ø BP 2152, Baltic Präparation, e.K., Wetter, Germany) using double-sided adhesive carbon discs and conductive adhesive paste (Leit-Tabs 12 mm Ø G 3347, BALTIC-Präparation e.K., Wetter, Germany). After photographic documentation (Macroscope M420, Wild, Heerbrugg, Germany; Axiocam 105 color, Carl Zeiss, Jena, Germany; 2560 × 1920 pixels, original magnification ×5.0), the sample surfaces were sputtered (BAL-TEC SCD 005, Balzers, Liechtenstein) with a platinum layer thickness of approximately 5 nm (platinum foil BP 2228, BALTIC-Präparation e.K., Wetter, Germany). The specimens were subsequently examined in a scanning electron microscope (FEI Quanta 400 FEG, FEI Company, ThermoFisher Scientific, FEI Deutschland GmbH, Frankfurt/Main, Germany; high vacuum mode, secondary electron mode, accelerating voltage 4 kV, spotsize 3, working distance 6–7 mm, aperture 30 µm ø). Ten micrographs per specimen in randomly chosen fields within a region revealing cellular growth in fluorescence microscopy were recorded at original magnification of ×30,000. For each cell line at least two independent samples were measured.

### 4.6. Superficial Energy Dispersive X-ray Spectroscopy (EDX)

In the same region revealing cellular growth in fluorescence microscopy per specimen, as described for HV-SEM-examination, the elemental composition in a centrally located field at original magnification of ×500 (horizontal field width 540 µm) was determined by EDX (EDAX Octane Elect Detector, APEX v2.0 software; Ametek EDAX GmbH, Weiterstadt, Germany). EDX analyses were performed in high vacuum mode (accelerating voltage 10 kV, Spotsize 5, working distance 10 mm, aperture 50 µm ø.) on platin-sputtered specimens as described before. Elemental mappings (smart phase mappings, tolerance adjustment 0.95, 256 × 200 pixels, 12,000–13,000 cps, amp time 1.92 µs, dwell time 200 µs, 230–250 cycles) and exemplary linescans along the surface morphologies (resolution 0.9 µm, line width 2 µm, dwell time 20 ms, 12,000–13,000 cps, amplification time = 7.68 µs, 30–35 cycles) were performed in the chosen areas. The presence of the phases of similar elemental composition, as detected by smart phase mapping, were calculated as the relative proportion of all pixels of the total area. Hereby, using a tolerance adjustment of 0.95, phases with different elemental elemental compositions were identified for one exemplary specimen per group. Subsequently, each pixel was assigned to one of the identified phases. For the exemplarily selected areas, the percentage of all pixels of the corresponding section was calculated for the respective phases. Furthermore, the relative elemental compositions of the corresponding phases were calculated in relative atomic percent (At%) for each detected element (C, N, O, Na, Mg, P, Ca, Pt).

### 4.7. Scoring of Samples

Visual scoring with 10 different samples each were performed independently and blindly by 3 raters (D.F., D.S., R.J.B). The scores were matched using the previously determined scoring table (see Figure 2). Exemplary raw images for scoring are shown in Supplementary Figure S2. Scorers had no other information except the example scoring images and the scoring Table 1.

### 4.8. Statistical Analysis

Statistical analyses were performed using GraphPad Prism 8 software (Version 8 of GraphPad Software, Inc., Boston, MA, USA). All assays were performed in replicates, and the results are presented as means ± SD. Two-tailed Student’s *t*-test, ratio paired *t*-tests or multiple *t*-tests were used for comparison between groups. *p* ≤ 0.05 was considered as statistically significant.

## 5. Conclusions

In conclusion, our study highlights the complex role of BMSCs in the OSCC tumor microenvironment and the potential impact of BMSC/HNC interactions on bone material composition and tumor invasion. Our findings emphasize the need for further investigation to identify novel therapeutic strategies targeting the interactions between tumor cells and stromal cells in HNC.

## Figures and Tables

**Table 1:** Scoring table for collagen exposure.

Grade	Collagen Exposure
1	No collagen exposure
2	<30% Collagen exposure
3	30–50% Collagen exposure
4	50–90% Collagen exposure
5	>90% Collagen exposure

## Data Availability

Data is contained within the article or Supplementary Material.

## Supplementary Materials

The following supporting information can be downloaded at: https://www.mdpi.com/article/10.3390/ijms241914417/s1.
