# Supplementary figures and images for: BMSC–HNC Interaction: Exploring Effects on Bone Integrity and Head and Neck Cancer Progression
Source: Int J Mol Sci. 2023 Sep 22;24(19):14417. doi: 10.3390/ijms241914417 (PMC10573008; doi:10.3390/ijms241914417)

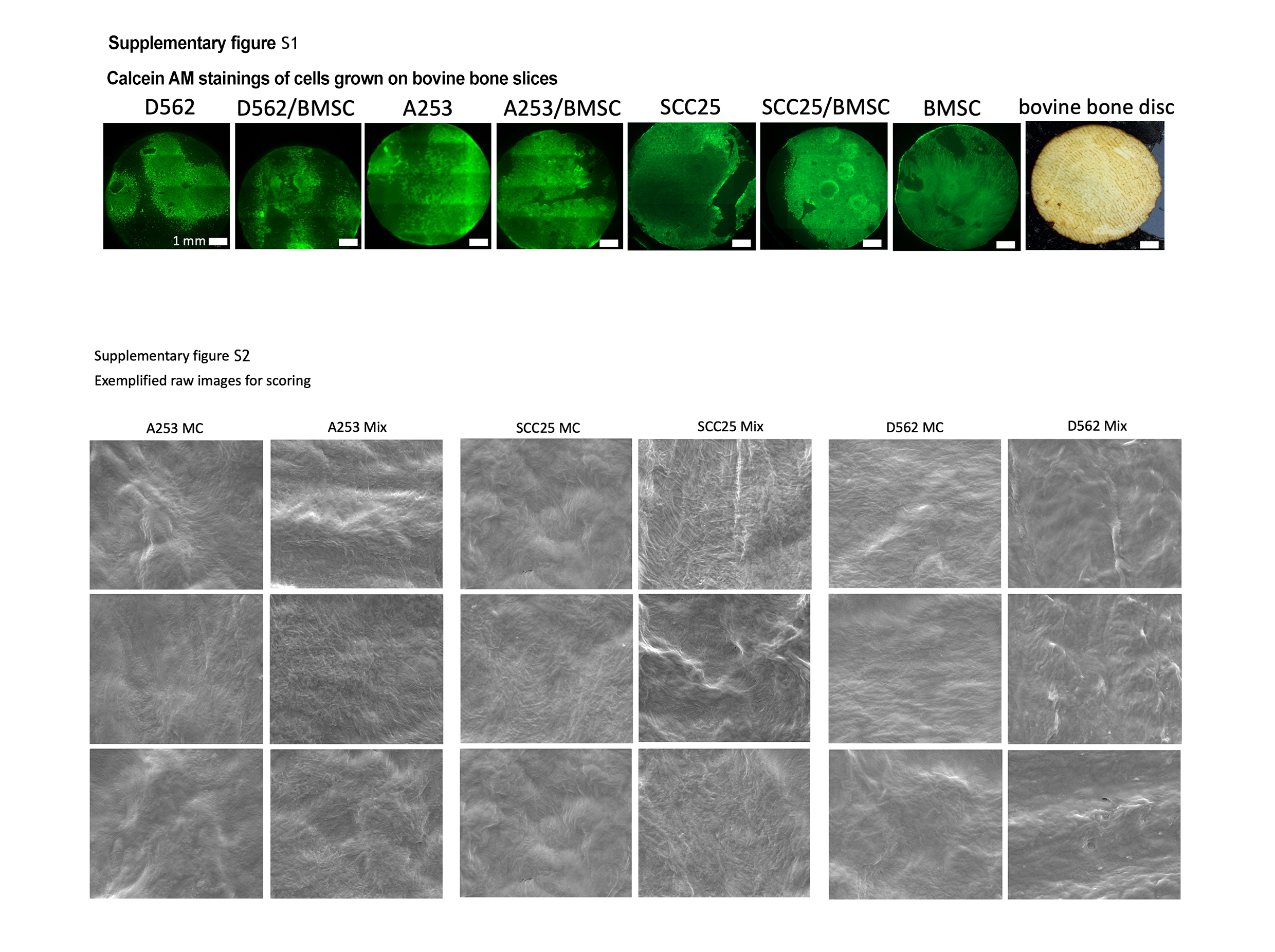

Supplement: Supplementary file 1 [file ijms-24-14417-s001.zip › ijms-2602994-supplementary.tif]
